# Substance use and pre-hospital crash injury severity among U.S. older adults: A five-year national cross-sectional study

**DOI:** 10.1371/journal.pone.0293138

**Published:** 2023-10-25

**Authors:** Oluwaseun Adeyemi, Marko Bukur, Cherisse Berry, Charles DiMaggio, Corita R. Grudzen, Sanjit Konda, Abidemi Adenikinju, Allison Cuthel, Jean-Baptiste Bouillon-Minois, Omotola Akinsola, Alison Moore, Ryan McCormack, Joshua Chodosh

**Affiliations:** 1 Ronald O Perelman Department of Emergency Medicine, New York University Grossman School of Medicine, New York, New York, United States of America; 2 Department of Surgery, New York University Grossman School of Medicine, New York, New York, United States of America; 3 Department of Population Health, New York University Grossman School of Medicine, New York, New York, United States of America; 4 Department of Medicine, Memorial Sloan Kettering Cancer Center, New York, New York, United States of America; 5 Department of Orthopedics, New York University Grossman School of Medicine, New York, New York, United States of America; 6 Department of Orthopedics, Mayo Clinic, Rochester, Minnesota, United States of America; 7 Emergency Department, CHU Clermont-Ferrand, Clermont-Ferrand, France; 8 Department of Social Work, Minnesota State University, Mankato, Minnesota, United States of America; 9 Department of Medicine, University of California San Diego, San Diego, California, United States of America; 10 Department of Medicine, New York University School of Medicine, New York, NY, United States of America; 11 Medicine Service, Veterans Affairs New York Harbor Healthcare System, New York, NY, United States of America; Southeast University, CHINA

## Abstract

**Background:**

Alcohol and drug use (substance use) is a risk factor for crash involvement.

**Objectives:**

To assess the association between substance use and crash injury severity among older adults and how the relationship differs by rurality/urbanicity.

**Methods:**

We pooled 2017–2021 cross-sectional data from the United States National Emergency Medical Service (EMS) Information System. We measured injury severity (low acuity, emergent, critical, and fatal) predicted by substance use, defined as self-reported or officer-reported alcohol and/or drug use. We controlled for age, sex, race/ethnicity, road user type, anatomical injured region, roadway crash, rurality/urbanicity, time of the day, and EMS response time. We performed a partial proportional ordinal logistic regression and reported the odds of worse injury outcomes (emergent, critical, and fatal injuries) compared to low acuity injuries, and the predicted probabilities by rurality/urbanicity.

**Results:**

Our sample consisted of 252,790 older adults (65 years and older) road users. Approximately 67%, 25%, 6%, and 1% sustained low acuity, emergent, critical, and fatal injuries, respectively. Substance use was reported in approximately 3% of the population, and this proportion did not significantly differ by rurality/urbanicity. After controlling for patient, crash, and injury characteristics, substance use was associated with 36% increased odds of worse injury severity. Compared to urban areas, the predicted probabilities of emergent, critical, and fatal injuries were higher in rural and suburban areas.

**Conclusion:**

Substance use is associated with worse older adult crash injury severity and the injury severity is higher in rural and suburban areas compared to urban areas.

## 1. Introduction

Every day in the United States (U.S.), approximately 700 older adults sustain crash injuries with varying degrees of severity [1]. As of 2020, there were over 44 million licensed older adult drivers in the U.S. ‐ a 68% increase compared to two decades ago (1). These older adult drivers are at increased crash risk due to low visual acuity, poor peripheral vision, presence of other eye diseases, hearing loss, decline in motor skills, and other environmental road conditions such as nighttime driving [2,3]. However, crash injuries involving older adults extend beyond being car occupants but include pedestrians, riders of bicycles and tricycles, and bus or truck occupants. While motor vehicular crashes account for about 55% of crash injuries among older adults [4,5], pedestrian crash rates (secondary to motor vehicle use) have also been on the rise, increasing from 40.7 to 45.0 per 100,000 population between 2009 and 2019 [6].

Further increasing the risk of crash involvement and injury among older adults is alcohol and drug use (collectively referred to as substance use) [7–10]. It is estimated that approximately 38,000 older adults receive opioid prescriptions every day, one out of every eight older adults takes alcohol daily, and a smaller proportion report daily use of marijuana and cocaine [11,12]. Across all age groups, substance use while driving is associated with 45% increased odds of adverse crash outcomes and increased risk of pre-hospital crash fatality [13,14]. Alcohol is associated with two to seven folds increased odds of crash involvement in a crash [15–17], and a 15-fold increased odds of severe injury [16]. Marijuana, opioids, narcotics, stimulants, and depressants are associated with two to six folds increased crash risks and odds of fatal crash injuries [18–23].

Although crash injuries are preventable, rapid provision of care can improve injury outcomes among older adults. The rural-urban disparity in Emergency Medical Service (EMS) response [24,25], may disproportionately predispose older adults with crash injuries to worse injury severity compared to older adults with similar injuries in urban areas [26]. Earlier studies have reported that, while fatal injuries occur more in rural areas, minor and serious injuries occur more in urban areas [14,27,28]. Additionally, substance use differs across rurality/urbanicity. While urban areas have a higher incidence of hallucinogens, cocaine, marijuana, and other illicit drug use, rural areas have a higher incidence of alcohol and opioid misuse [29].

It is unknown to what extent substance use is associated with injury severity among older adults. Additionally, it is not known how the relationship between substance use and crash injury severity among older adults differs across rural and urban areas. Identifying these regional differences may inform policies on safe driving, road infrastructural design, and targeted behavioral interventions for older adults. Assessing the risk of crash injury severity among older adults is important due to the increasing older US adult population [30,31], and older licensed drivers [1]. This study, therefore, aims to assess the relationship between substance use and crash injury severity among older adults and the rural-urban differences that further define this problem.

## 2. Methods

### 2.1. Study design

We conducted a cross-sectional analysis by pooling five years of data (2017 to 2021) from the National Emergency Medical Services (EMS) Information System (NEMSIS). The NEMSIS is the national database of all EMS cases across U.S. States and territories [32]. Between 2017 and 2021, the number of states and territories that reported their EMS statistics to NEMSIS and permitted its use for research increased from 35 to 53 and the number of 9-1-1 events captured in the NEMSIS data increased from 7,907,829 to 48,982,990 [33].

### 2.2. Inclusion and exclusion criteria

**Fig 1:**
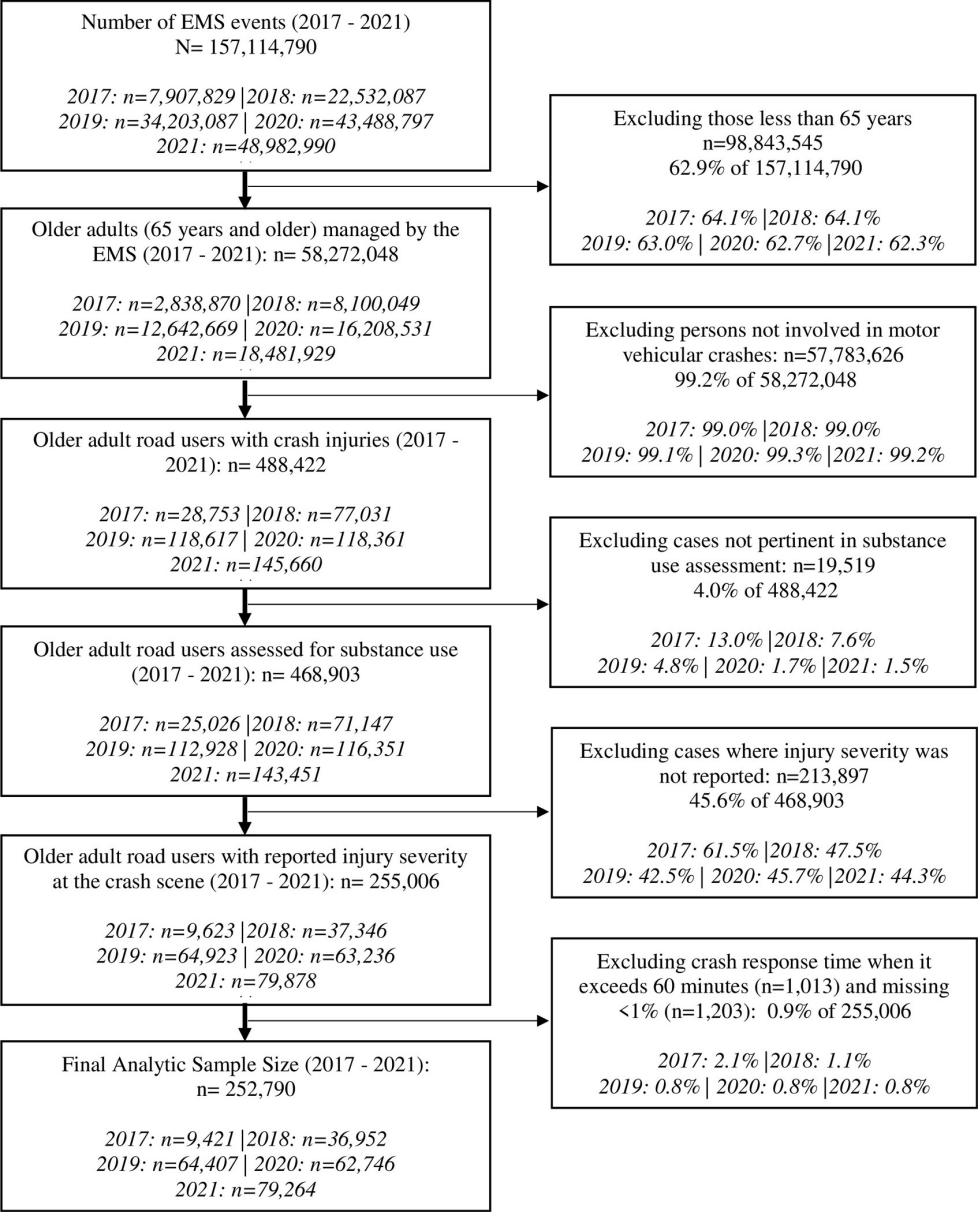
Data selection steps using the 2017 to 2021 National Emergency Medical Service (EMS) Information System database.

Between 2017 and 2021, 157,114,790 persons were managed following an EMS activation (Fig 1). We identified the older adult population (age 65 years and older) (n = 58,272,048). We further restricted the population to age 65 years and older road users that sustained motor vehicle crash injuries using the International Classification of Disease version 10 (ICD-10) codes V00 to V79 (n = 488,422). We excluded cases whose substance use status was coded as "not applicable" (n = 19,519; 4% of 488,422). Thereafter, we excluded cases whose injury status was not reported (n = 213,897; 45.6% of 468,903). These unreported cases represent patients who either canceled the 9-1-1 call, refused care, or were evaluated but no treatment or transport was required. Also, we performed a listwise deletion for cases whose missingness was less than one percent (n = 1,203; 0.5% of 255,006) and when the crash response time was greater than 60 minutes (n = 1,103; 0.4% of 255,006). We excluded cases whose EMS response time exceeded 60 minutes, consistent with an earlier study [26]. These outlier cases are typically associated with unique environmental conditions such as tornadoes [34–36]. The final analytic data, therefore, was a total of 252,790 older adult road users who sustained motor vehicle injuries.

### 2.3. Injury severity

Our outcome measure, injury severity status, is a four-point categorical variable: low acuity, emergent, critical, and fatal injury [37]. Patients with low acuity injuries have injuries with a low probability of worsening or developing serious complications in the absence of intervention. Patients with emergent injuries have the potential of worsening if intervention is not initiated quickly. Patients with critical injuries have life-threatening injuries at high mortality risk if intervention is not initiated immediately. Finally, those with fatal injuries either died at the crash scene or while in transit to the hospital. These categorizations were made by EMS providers at the crash scene using the Model of the Clinical Practice of Emergency Medicine [38,39].

### 2.4. Substance use

The main predictor variable is the presence or absence of substance use. We defined substance use using the variable eHistory.17, representing alcohol/drug use indicators [40]. The NEMSIS defines substance use in seven categories: 1) alcohol containers/paraphernalia at the scene, 2) drug paraphernalia at the scene, 3) patient admits to alcohol use, 4) patient admits to drug use, 5) positive level (of alcohol or drugs) known from law enforcement or hospital record, 6) smell of alcohol on breath, 7) none reported. We defined the presence of substance use at the time of the crash event as cases in the first to sixth categories while the absence of substance use at the time of the crash was defined with cases coded in the seventh category [40]. Hence, an individual who is positive for substance use either used alcohol, drugs, or both. In the NEMSIS documentation, an individual can be assigned multiple categories. We, therefore, defined cases of “alcohol use only” using categories 1, 3, 5, and 6 and “drug use only” using categories 2 and 4.

### 2.5. Patient and injury characteristics

We controlled for age, sex, race/ethnicity, road user type, anatomical injured region, rurality/urbanicity, roadway location of the crash, the time of the day of the crash, and the EMS response time. We selected these patient and injury characteristics a priori from the literature [14,24,26]. Age was measured as a three-level categorical variable (65–74 years, 75–84 years, and 85 years and older), consistent with earlier definitions of phases of aging [41–43]. Sex was measured as a binary variable. We defined race/ethnicity in four categories: non-Hispanic White, non-Hispanic Black, Hispanic, and other races. Road user type was measured in six categories using the ICD-10 codes: car occupants (V40 –V49), pedestrians (V00 –V09), two-wheel vehicle occupants (V10 –V29), three-wheel vehicle occupants (V30 –V39), occupants of buses (V70 –V79), and occupants of trucks and industrial vehicles (V50 –V69). We defined the injured anatomic region in five categories: injury to the head and neck, abdomen and genitals, chest and back, upper and lower limbs (extremities), and multiple body injuries. NEMSIS reports the geographical location as a four-point categorical variable: wilderness, rural, suburban, and urban, using the United States Department of Agriculture Urban influence codes [37]. We recoded this variable into three categories: rural/wilderness (hereafter referred to as rural), suburban, and urban.

We assessed whether whether the older adult road user had a roadway crash or not. Using the ICD-10 codes, we identified roadway crash as crash events that occurred on street, highway and other paved roadways (Y92.4). All other location, which includes places of residence, businesses, stores, recreational areas, schools, and places not otherwise specified, were classified as not a roadway crash. We defined the time of the crash injury using a proxy measure–the time the 9-1-1 call was initiated. The time of crash injury was defined in four categories: morning rush hour period, afternoon rush hour period, nighttime, and other hours. Using a recently published meta-analysis as a guide [44], the morning and afternoon rush hour periods were defined as crash injuries sustained between 6 to 9 am and 3 to 7 pm, respectively. Nighttime crashes were defined as crash injuries occurring between 12 midnight and 5 am. We defined the EMS response time as the duration from chute initiation at the base station to arrival at the crash scene. We measured the crash response time as a four-point categorical variable: less than nine minutes, nine to 17.59 minutes, 18 to 26.59 minutes, and 27 minutes or higher. The nine-minute benchmark is based on the guidelines of the National Fire Protection Agency and the Fire and EMS Department, which requires the EMS travel time to be less than 9 minutes [45,46].

### 2.6. Handling of missing data

We encountered missing values in the following variables: substance use (18.0%), race/ethnicity (30.5%), roadway crash (1.6%), and anatomical injured region (25.7%). We performed multiple imputations for missing data, using the multiple imputations with chained equation (MICE) after justifying that missingness was at random [47]. Additionally, NEMSIS had advised researchers not to assume that missingness in the NEMSIS data is “Not Missing at Random” [48], further stressing the need to perform some measures of missing data analysis whenever such missingness is encountered. The MICE model was strengthened using injury severity, age, sex, crash response time, road user type, and the time of the crash as predictors. We performed 100 iterations, generated 100 predicted values for all missing values, and assigned the final value using the mean of the predicted values, consistent with earlier literature on multiple imputations [49,50].

### 2.7. Analysis

We computed the frequency distribution of demographic, injury, crash, and substance use characteristics. We assessed differences across injury severity status and rurality/urbanicity using chi-square statistics. We performed unadjusted and adjusted partially proportional ordinal logistic regression [51] to assess the odds of worse injury outcomes (critical, emergent, and fatal injuries) and computed the predicted probabilities of substance use-associated injury severity. The decision to use a partially proportional ordinal logistic regression, as opposed to a proportionally ordinal regression was based on the violation of the parallel lines assumption evidenced by a significant Brant test [51]. Also, we performed the interaction analysis between substance use and rurality/urbanicity and we reported the predicted probabilities of each substance use-related injury severity category in rural, suburban, and urban areas. Data were analyzed using SAS 9.4 [52] and STATA version 17 [53].

### 2.8. Ethical concern

This research used the 2017 ‐ 2021 NEMSIS data, publicly available de-identified data [54]. Based on the guidance from the New York University Langone Health Institutional Review Board (IRB), secondary data analysis of de-identified data that is publicly available does not require IRB approval [55]. Hence, informed consent was not required for this study. Also, since the de-identified data is available publicly and obtainable upon request from NEMSIS, our secondary data analysis was adjudged as not human subject research [55]. Our study followed the STROBE guidelines for reporting cross-sectional studies (available as a supporting information file).

## 3. Results

A total of 252,790 older adults met our inclusion and exclusion criteria (Table 1). The majority of the population was between 65 and 74 years (62%), female (51%), non-Hispanic Whites (71%), and car occupants (76%). Thirty-six percent of the sample population sustained injuries to the chest and back. The crash injuries occurred mostly in urban areas (83%) and were mostly roadway crashes (80%), with 16% of the crash events occurring during the afternoon rush hour period. Approximately 75% of the older adults experienced an EMS response time of less than nine minutes. Substance use was identified in approximately 3% of the sample population with cases of only alcohol and only drug impairments being 2.8% and 0.4%, respectively. Furthermore, 67% of the sample population had low acuity injuries, 25%, and 6% sustained emergent and critical injuries and 1% died. Age, sex, race/ethnicity, road user type, the anatomical injured region, geographical location, roadway crash, time of the day, EMS response time, and measures of substance use were significantly associated with pre-hospital crash injury severity (p<0.001).

**Table 1:** Frequency distribution and summary statistics of the demographic, crash, injury, and substance use characteristics of the study population (N=252,790).

Variables	Frequency (%) (N = 252,790)	Low Acuity n = 170,165 (67.3%)	Emergent n = 63,864 (25.3%)	Critical n = 15,959 (6.3%)	Fatal n = 2,802 (1.1%)	p-value*
Age Categories						
65–74 years	156,866 (62.0)	106,523 (62.6)	39,119 (61.3)	9,541 (59.8)	1,683 (60.1)	<0.001
75–84 years	72,602 (28.7)	48,785 (28.7)	18,588 (29.1)	4,440 (27.8)	789 (28.2)	
85 years and older	23,322 (9.2)	14,857 (8.7)	6,157 (9.6)	1,978 (12.4)	330 (11.7)	
Sex						
Male	124,180 (49.1)	77,467 (45.5)	34,685 (54.3)	10,137 (63.5)	1,891 (67.5)	<0.001
Female	128,610 (50.9)	92,698 (54.5)	29,179 (45.7)	5,822 (36.5)	911 (32.5)	
Race/Ethnicity						
Non-Hispanic White	180,552 (71.4)	115,833 (68.1)	49,464 (77.4)	12,938 (81.1)	2,317 (82.7)	<0.001
Non-Hispanic Black	53,866 (21.3)	41,195 (24.2)	10,329 (16.2)	2,012 (12.6)	330 (11.8)	
Hispanic	12,951 (5.1)	9,293 (5.4)	2,820 (4.4)	708 (4.4)	130 (4.6)	
Other Races	5,421 (2.2)	3,844 (2.3)	1,251 (2.0)	301 (1.9)	25 (0.9)	
Road User Type						
Car Occupant	193,325 (76.1)	137,191 (80.6)	45,022 (70.5)	9,399 (58.9)	1,713 (61.1)	<0.001
Pedestrian	24,921 (9.8)	13,876 (8.1)	7,645 (12.0)	2,898 (18.2)	502 (17.9)	
Two Wheel Vehicle Occupant	23,216 (9.2)	12,343 (7.2)	7,998 (12.5)	2,599 (16.3)	276 (9.8)	
Three Wheel Vehicle Occupant	352 (0.1)	173 (0.1)	132 (0.2)	44 (0.3)	3 (0.1)	
Bus Occupant	1,595 (0.6)	1,170 (0.7)	332 (0.5)	75 (0.5)	18 (0.6)	
Truck/Industrial Vehicle Occupant	9,381 (3.7)	5,412 (3.2)	2,735 (4.3)	944 (5.9)	290 (10.2)	
Anatomical Injured Region						
Head and Neck	51,730 (20.5)	29,868 (17.6)	16,228 (25.4)	4,996 (31.3)	638 (22.8)	<0.001
Chest and Back	91,533 (36.2)	69,680 (41.0)	19,247 (30.1)	2,496 (15.7)	110 (3.9)	
Abdomen and Genitals	4,574 (1.8)	2,770 (1.6)	1,403 (2.2)	390 (2.4)	11 (0.4)	
Extremities	41,302 (16.3)	29,830 (17.5)	9,832 (15.4)	1,602 (10.0)	38 (1.4)	
Multiple Body Injuries	63,651 (25.2)	38,017 (22.3)	17,154 (26.9)	6,475 (40.6)	2,005 (71.5)	
Geographical Location						
Urban	208,917 (82.6)	144,015 (84.6)	50,922 (79.7)	12,037 (75.4)	1,943 (69.3)	<0.001
Suburban	20,176 (8.0)	12,584 (7.4)	5,365 (8.4)	1,855 (11.6)	408 (14.6)	
Rural	23,697 (9.4)	13,602 (8.0)	7,577 (11.9)	2,067 (13.0)	451 (16.1)	
Roadway Crash						
Yes	202,554 (80.1)	138,605 (81.4)	49,429 (77.4)	12,004 (75.2)	2,516 (89.8)	<0.001
No	50,236 (19.9)	31,560 (18.6)	14,435 (22.6)	3,955 (24.8)	286 (10.2)	
Time of the Day						
Morning Rush Hour	16,605 (6.6)	11,433 (6.7)	3,941 (6.2)	1,022 (6.4)	209 (7.5)	<0.001
Afternoon Rush Hour	40,578 (16.1)	27,428 (16.1)	10,077 (15.8)	2,666 (16.7)	407 (14.5)	
Nighttime Driving	11,084 (4.4)	7,206 (4.2)	2,902 (4.5)	793 (5.0)	183 (6.6)	
Other Hours	184,523 (73.0)	124,098 (72.9)	46,944 (73.5)	11,478 (71.9)	2,003 (71.5)	
EMS Response Time						
0–8.59 minutes	189,883 (75.1)	129,692 (76.2)	47,114 (73.8)	11,143 (69.8)	1,934 (69.0)	
9–17.59 minutes	50,205 (19.9)	33,105 (19.5)	12,891 (20.2)	3,497 (21.9)	712 (25.4)	
18–26.59 minutes	8,993 (3.6)	5,344 (3.1)	2,630 (4.1)	901 (5.7)	118 (4.2)	
27 minutes or higher	3,709 (1.5)	2,024 (1.2)	1,229 (1.9)	418 (2.6)	38 (1.4)	
Substance Use						
Yes	7,884 (3.1)	4,304 (2.5)	2,635 (4.1)	876 (5.5)	69 (2.5)	<0.001
No	245,906 (96.9)	165,861 (97.5)	61,229 (95.9)	15,083 (94.5)	2,733 (97.5)	
Alcohol Use Alone						
Yes	7,183 (2.8)	3,942 (2.3)	2,400 (3.8)	782 (4.9)	59 (2.1)	<0.001
No	245,607 (97.2)	166,223 (97.7)	61,464 (96.2)	15,177 (95.1)	2,743 (97.9)	
Drug Use Alone						
Yes	961 (0.4)	592 (0.4)	270 (0.4)	89 (0.6)	10 (0.4)	<0.001
No	251,829 (99.6)	169,573 (99.6)	63,594 (99.6)	15,870 (99.4)	2,792 (99.6)	

*Statistical differences in low acuity, emergent, and critical categories across all variables except Revised Trauma Score were assessed using chi-square

**Mean differences in Revised Trauma Score were assessed using the one-way ANOVA, and median differences were assessed using the Kruskal-Wallis test

***Special vehicles defined as three wheels, vehicles in other land transport, or other crash accidents not specified, corresponding to ICD 10 codes V30-39, and V80-99.

**Fig 2:**
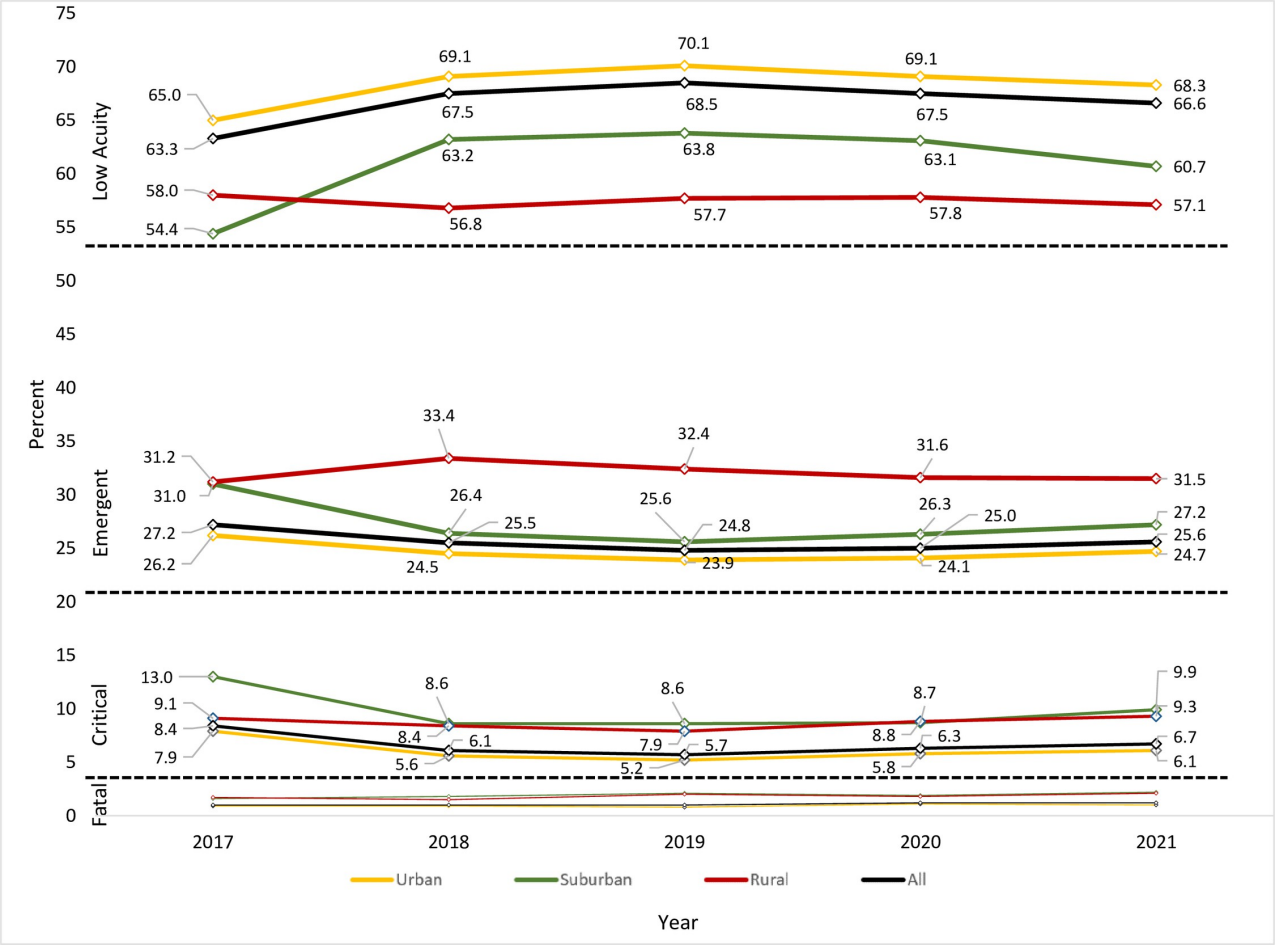
Trend of the proportion of low acuity, emergent, critical, and fatal crash injuries among older adult road users across rural, suburban, and urban areas between 2017 and 2021.

Between 2017 and 2021, the proportion of emergent injuries ranged from 24.8–27.2%, while the proportion of critical and fatal injuries ranged between 5.7% ‐ 8.4%, and 1.0 ‐ 1.2%, respectively (Fig 2). Among older adults with emergent injuries, the proportions in urban areas ranged between 23.9 ‐ 26.2%, while the proportions in suburban and rural areas ranged between 25.6–31.0%, and 31.2 ‐ 33.4%, respectively (p<0.001). Also, among older adults with critical injuries, the proportions ranged between 5.2 ‐ 7.9% in urban areas, and in suburban and rural areas, the proportions ranged between 8.6 ‐ 13.0% and 7.9 ‐ 9.3%, respectively (p<0.001). Furthermore, among older adults with fatal injuries, the proportions in urban areas ranged between 0.8 ‐ 1.1%, and in suburban and rural areas, the proportions ranged between 1.8 ‐ 2.2%, and 1.5 ‐ 2.1%, respectively (p<0.001). While there was a decline in emergent and critical injuries between 2017 and 2019, the proportions of these injuries gradually increased from 2019 to 2021 in rural, suburban, and urban areas (p<0.001).

There were significant differences in the age and sex of older adults who sustained crash injuries in rural, suburban, and urban areas (Table 2). The proportion of non-Hispanic Whites who sustained crash injuries in urban areas was 68% while the proportions in suburban and rural areas were 86% and 87%, respectively (p<0.001). While the proportion of pedestrians with crash injuries was highest in urban areas (urban– 10%, suburban– 8%, rural– 9%), the proportion of crashes among occupants of trucks and industrial vehicles was highest in rural areas (urban– 3%, suburban– 7%, rural– 8%) (p<0.001). The proportion of older adults who sustained multiple body injuries was 25% in urban areas, and in suburban and rural areas, the proportions were 28% and 29%, respectively (p<0.001). The proportion of roadway crashes was 81% in urban areas, and 76% and 75% in suburban and rural area, respectively (p<0.001). The proportion of older adults who experienced EMS response time of less than nine minutes was 77% in urban areas, and in suburban and rural areas, the proportions were 67% and 63%, respectively (p<0.001). Although the proportion of drug use in urban areas was significantly higher compared to suburban and rural areas (p<0.001), there were no differences in alcohol use in urban, suburban, and rural areas.

**Table 2:** Rural-urban differences in the frequency distribution and summary statistics of the demographic, crash, injury, and substance use characteristics of the study population (N=252,790).

Variables	Urban (n = 208,917)	Suburban (n = 20,176)	Rural (n = 23,697)	p-value*
Age Categories				
65–74 years	130,108 (62.3)	12,328 (61.1)	14,430 (60.9)	<0.001
75–84 years	59,486 (28.5)	6,041 (29.9)	7,075 (29.9)	
85 years and older	19,323 (9.2)	1,807 (9.0)	2,192 (9.2))	
Sex				
Male	101,217 (48.5)	10,452 (51.8)	12,511 (52.8)	<0.001
Female	107,700 (51.5)	9,724 (48.2)	11,186 (47.2)	
Race/Ethnicity				
Non-Hispanic White	142,675 (68.3)	17,331 (85.9)	20,546 (86.7)	<0.001
Non-Hispanic Black	49,681 (23.8)	2,025 (10.1)	2,160 (9.1)	
Hispanic	11,666 (5.6)	593 (2.9)	692 (2.9)	
Other Races	4,895 (2.3)	227 (1.1)	299 (1.3)	
Road User Type				
Car Occupant	161,057 (77.1)	14,895 (73.8)	17,373 (73.3)	<0.001
Pedestrian	21,124 (10.1)	1,607 (8.0)	2,190 (9.2)	
Two Wheel Vehicle Occupant	19,114 (9.1)	2,041 (10.1)	2,061 (8.7)	
Three Wheel Vehicle Occupant	231 (0.1)	52 (0.3)	69 (0.3)	
Bus Occupant	1,381 (0.7)	99 (0.5)	115 (0.5)	
Truck/Industrial Vehicle Occupant	6,010 (2.9)	1,482 (7.3)	1,889 (8.0)	
Anatomical Injured Region				
Head and Neck	43,116 (20.6)	3,902 (19.3)	4,712 (19.9)	<0.001
Chest and Back	77,770 (37.2)	6,558 (32.5)	7,205 (30.4)	
Abdomen and Genitals	3,669 (1.8)	428 (2.1)	477 (2.0)	
Extremities	33,025 (15.8)	3,721 (18.5)	4,556 (19.2)	
Multiple Body Injuries	51,337 (24.6)	5,567 (27.6)	6,747 (28.5)	
Roadway Crash				
Yes	169,390 (81.1)	15,353 (76.1)	17,811 (75.2)	<0.001
No	39,527 (18.9)	4,823 (23.9)	5,886 (24.8)	
Time of the Day				
Morning Rush Hour	14,060 (6.7)	1,039 (5.2)	1,506 (6.4)	<0.001
Afternoon Rush Hour	33,777 (16.2)	3,195 (15.8)	3,606 (15.2)	
Nighttime Driving	9,198 (4.4)	870 (4.3)	1,016 (4.3)	
Other Hours	151,882 (72.7)	15,072 (74.7)	17,569 (74.1)	
EMS Response Time				
0–8.59 minutes	161,366 (77.2)	13,486 (66.8)	15,031 (63.4)	<0.001
9–17.59 minutes	39,407 (18.9)	4,559 (22.6)	6,239 (26.3)	
18–26.59 minutes	5,996 (2.9)	1,328 (6.6)	1,669 (7.1)	
27 minutes or higher	2,148 (1.0)	803 (4.0)	758 (3.2)	
Substance Use				
Yes	6,568 (3.1)	609 (3.0)	707 (3.0)	0.281
No	202,349 (96.9)	19,567 (97.0)	22,990 (97.0)	
Alcohol Use Alone				
Yes	5,993 (2.9)	550 (2.7)	640 (2.7)	0.199
No	202,924 (97.1)	19,626 (97.3)	23,057 (97.3)	
Drug Use Alone				
Yes	849 (0.4)	53 (0.3)	59 (0.3)	<0.001
No	208,068 (99.6)	20,123 (99.7)	23,638 (99.7)	

*Statistical differences in rural, suburban, and urban categories across all variables except Revised Trauma Score were assessed using chi-square

**Mean differences in Revised Trauma Score were assessed using the one-way ANOVA, and median differences were assessed using the Kruskal-Wallis test.

Compared to low acuity injury, the unadjusted odds of worse injury severity were higher with increasing age, among males, pedestrians, occupants of two and three-wheeled vehicles, and occupants of trucks and industrial vehicles (Table 3). Worse injury severity was also higher among those with injuries to the head and neck, those that were involved in nighttime driving, and with increasing EMS crash response time. Compared to injuries in urban areas, injuries that occurred in rural (OR: 1.65; 95% CI: 1.60 ‐ 1.69) and suburban areas (OR: 1.35; 95% CI: 1.31 ‐ 1.39) were associated with increased odds of worse injury severity. Substance use was associated with 1.7 (95% CI: 1.67 ‐ 1.83) times the odds of worse injury severity. After adjusting for potential confounders, substance use was associated with 1.36 times the adjusted odds of worse injury severity (95% CI: 1.30 ‐ 1.43). Alcohol use alone was associated with 1.33 (95% CI: 1.27 ‐ 1.40) times the adjusted odds of worse injury severity while drug use alone was associated with 1.15 (95% CI: 1.00–1.32) times the adjusted odds of worse injury severity.

**Table 3:** Unadjusted and adjusted odds ratio of worse injury severity (critical, emergent, death vs. low acuity) associated with the demographic, crash, injury, and substance use characteristics among older adults.

Variables	Emergent/Critical/Fatal vs. Low Acuity			
	Univariate Model	Substance Use Model	Alcohol Alone Model	Drugs Alone Model
	Unadjusted Odds Ratio (95% CI)	Adjusted Odds Ratio (95% CI)***	Adjusted Odds Ratio (95% CI)***	Adjusted Odds Ratio (95% CI)***
Substance Use				
Yes	**1.75 (1.67–1.83)**	**1.36 (1.30–1.43)**		
No	Ref	Ref		
Alcohol Use Alone				
Yes	**1.72 (1.64–1.80)**		**1.33 (1.27–1.40)**	
No	Ref		Ref	
Drug Use Alone				
Yes	**1.28 (1.13–1.46)**			**1.15 (1.00–1.32)**
No	Ref			Ref
Age Categories				
85 years and older	**1.21 (1.17–1.24)**	**1.19 (1.15–1.22)**	**1.19 (1.15–1.22)**	**1.18 (1.14–1.21)**
75–84 years	**1.03 (1.01–1.05)**	**1.05 (1.03–1.07)**	**1.05 (1.02–1.07)**	**1.04 (1.02–1.06)**
65–74 years	Ref	Ref	Ref	Ref
Sex				
Male	**1.56 (1.53–1.58)**	**1.29 (1.26–1.31)**	**1.29 (1.27–1.31)**	**1.30 (1.28–1.32)**
Female	Ref	Ref	Ref	Ref
Race/Ethnicity				
Non-Hispanic Black	**0.55 (0.54–0.56)**	**0.60 (0.59–0.62)**	**0.60 (0.59–0.62)**	**0.60 (0.59–0.62)**
Hispanic	**0.70 (0.68–0.73)**	**0.72 (0.69–0.75)**	**0.72 (0.69–0.75)**	**0.72 (0.69–0.75)**
Other Races	**0.73 (0.69–0.78)**	**0.77 (0.72–0.82)**	**0.77 (0.72–0.82)**	**0.77 (0.72–0.82)**
Non-Hispanic White	Ref	Ref	Ref	Ref
Road User Type				
Pedestrian	**1.95 (1.89–2.00)**	**1.94 (1.89–2.00)**	**1.94 (1.89–2.00)**	**1.95 (1.90–2.01)**
Two Wheel Occupant	**2.15 (2.09–2.21)**	**1.97 (1.92–2.03)**	**1.97 (1.92–2.03)**	**1.98 (1.92–2.04)**
Three Wheel Occupant	**2.53 (2.05–3.12)**	**2.35 (1.89–2.91)**	**2.34 (1.89–2.91)**	**2.35 (1.89–2.91)**
Bus Occupant	**0.89 (0.79–0.99)**	0.97 (0.86–1.08)	0.97 (0.86–1.08)	0.96 (0.86–1.08)
Truck/Industrial Vehicle Occupant	**1.79 (1.72–1.87)**	**1.56 (1.49–1.63)**	**1.56 (1.49–1.63)**	**1.56 (1.49–1.63)**
Car Occupant	Ref	Ref	Ref	Ref
Anatomical Injured Region				
Head and Neck	**1.09 (1.06–1.11)**	**1.13 (1.10–1.16)**	**1.13 (1.10–1.16)**	**1.13 (1.10–1.16)**
Chest and Back	**0.47 (0.46–0.48)**	**0.49 (0.48–0.50)**	**0.49 (0.48–0.50)**	**0.48 (0.47–0.49)**
Abdomen and Genitals	0.97 (0.91–1.03)	1.01 (0.95–1.07)	1.01 (0.95–1.07)	1.00 (0.94–1.07)
Extremities	**0.57 (0.56–0.59)**	**0.50 (0.49–0.52)**	**0.50 (0.49–0.52)**	**0.50 (0.49–0.52)**
General Body	Ref	Ref	Ref	Ref
Geographical Location				
Rural	**1.65 (1.60–1.69)**	**1.44 (1.40–1.48)**	**1.44 (1.40–1.48)**	**1.44 (1.40–1.48)**
Suburban	**1.35 (1.31–1.39)**	**1.20 (1.16–1.23)**	**1.19 (1.16–1.23)**	**1.19 (1.16–1.23)**
Urban	Ref	Ref	Ref	Ref
Roadway Crash				
Yes	**0.78 (0.76–0.80)**	**0.93 (0.91–0.95)**	**0.93 (0.91–0.95)**	**0.93 (0.91–0.95)**
No	Ref	Ref	Ref	Ref
Time of the Day				
Morning Rush Hour	**0.93 (0.90–0.96)**	**0.91 (0.88–0.95)**	**0.91 (0.88–0.95)**	**0.90 (0.88–0.94)**
Afternoon Rush Hour	0.98 (0.96–1.01)	**0.97 (0.95–0.99)**	**0.97 (0.95–0.99)**	**0.97 (0.95–0.99)**
Nighttime Driving	**1.11 (1.06–1.15)**	**1.06 (1.02–1.11)**	**1.06 (1.02–1.11)**	**1.07 (1.02–1.11)**
Other Hours	Ref	Ref	Ref	Ref
EMS Response Time				
27 minutes or higher	**1.79 (1.68–1.91)**	**1.43 (1.34–1.53)**	**1.43 (1.34–1.53)**	**1.43 (1.33–1.53)**
18–26.59 minutes	**1.47 (1.41–1.54)**	**1.29 (1.24–1.35)**	**1.29 (1.24–1.35)**	**1.29 (1.24–1.35)**
9–17.59 minutes	**1.11 (1.09–1.14)**	**1.07 (1.04–1.09)**	**1.07 (1.04–1.09)**	**1.07 (1.04–1.09)**
0–8.59 minutes	Ref	Ref	Ref	Ref

*Univariate logistic regression was used to perform the analysis

**Partially proportional ordinal logistic regression was used to perform the analysis

***Adjusted model consists of all listed variables.

**Fig 3:**
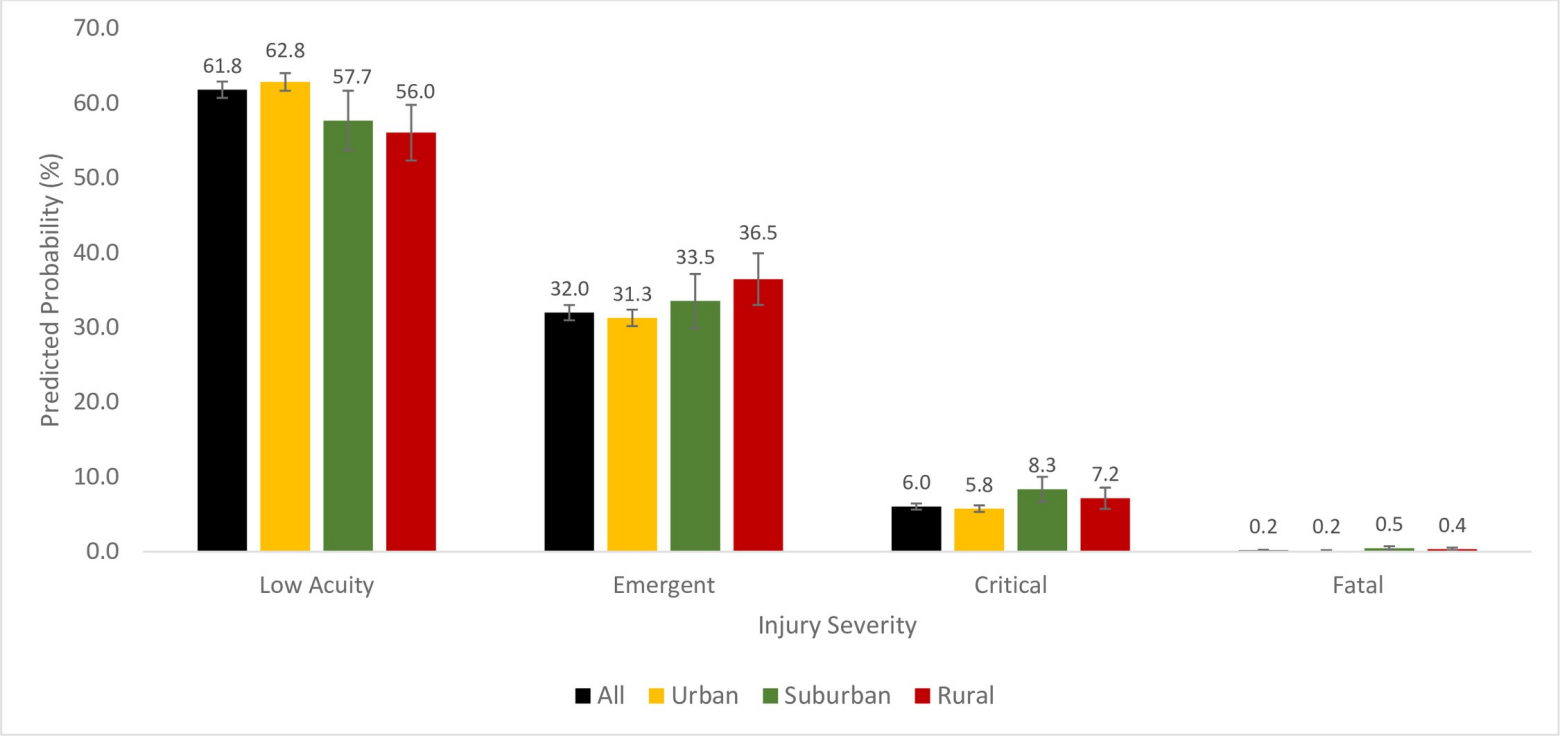
Predicted probabilities of low acuity, emergent, critical, and fatal crash injuries among older adult road users in all areas, and rural, suburban, and urban areas.

The predicted probability of substance use-associated emergent injury was 32.0% (95% CI: 31.0 ‐ 33.0) and the predicted probability increased step-wisely from urban (31.2%; 95% CI: 30.1 ‐ 32.3) to suburban (33.6; 95% CI: 30.0 ‐ 37.3) and rural areas (36.9%; 95% CI: 33.4 ‐ 40.4) (p<0.001) (Fig 3). Also, the predicted probability of substance use-associated critical injury was 6.0% (95% CI: 5.6 ‐ 6.5). The predicted probability was lowest in urban areas (5.8%; 95% CI: 5.3 ‐ 6.2) and in suburban and rural areas, the values were 8.4% (95% CI: 6.7 ‐ 10.0) and 7.1% (95% CI: 5.7 ‐ 8.5), respectively (p<0.001). Furthermore, the predicted probability of substance use-associated fatal injury was 0.2% (95% CI: 0.1 ‐ 0.2). The predicted probability was 0.2% (95% CI: 0.1 ‐ 0.2) in urban areas, and in suburban and rural areas, the values were 0.5% (95% CI: 0.2 ‐ 0.7) and 0.4% (95% CI: 0.2 ‐ 0.6), respectively (p<0.001).

## 4. Discussion

We present one of the few studies that demonstrate an association between substance use and crash injury severity among older adult road users. Also, our study provides a statistical basis for the conceptual linkage between substance use and injury severity among older adult road users, and how this association vary by rurality/urbanicity. Additionally, the observation that the predicted probabilities of substance use-related severe injuries are higher in rural and suburban areas compared to urban areas despite no rural-urban differences in the proportions of substance use among older adult road users highlight the rural-urban disparity in crash outcomes. Furthermore, the uptrend pattern in the proportions of emergent, critical, and fatal injuries between 2019 and 2021 in rural, suburban, and urban areas requires urgent public health intervention.

Earlier studies have reported the harmful effect of alcohol and drug use among older adults some of which include increased risk of falls [56–58], cognitive impairment [59,60], increased risk of alcohol or drug dependence [61,62], and worsening health conditions [63–65]. Our report of the increased odds of worse injury severity from substance use adds to the extant literature on the harms associated with alcohol and/or drug use. The worse injury outcomes associated with substance use among older adult road users may be explained by the reduced efficiency in metabolizing alcohol and drugs, longer toxic exposure, and an attenuation of the injury response mechanism when older adults are exposed to acute and/or chronic use of alcohol or drugs [66]. Acute and chronic alcohol use is associated with orthostatic hypotension and hypertension [67–69], respectively, and drugs such as opioids and benzodiazepines are central nervous system depressants [70,71]. Alcohol and drugs may further impair age-related physiologic response to acute trauma, hence increasing injury morbidity among older adults.

Similar to earlier studies that reported no rural-urban differences in alcohol and drug consumption [72,73], we report that there are no rural-urban differences in substance use-related crash injuries among older adult road users. However, there are significant rural-urban differences in the injury severity among older adults with substance use-related crash injuries. While the predicted probabilities of low acuity injuries decreased from urban to suburban and rural areas, the predicted probabilities of emergent injuries increased step-wise from urban to suburban and rural areas. Additionally, suburban and rural areas had higher critical and fatal injury probabilities compared to urban areas. These observed variations may reflect the rural-urban differences in driving behavior and access to timely and appropriate emergency care. Earlier studies have reported increased speeding behavior among road users in rural areas [74] and non-use of seat belts among older adult drivers [75]. Rural areas also experience significantly prolonged response times [24,26], increased deaths at the crash scene [26], and increased proportions of crash fatalities [76–78]. Additionally, the impact of hospital closures in rural areas may further explain the rural-urban disparity in crash outcomes since hospital closures is directly linked with reduced availaibility of healthcare professionals and reduced access to healthcare [79–82].

Crash injuries among older adult road users are preventable and interventions aimed at reducing substance use among road users represent a strategy for reducing crash-related morbidity and mortality. Excluding crash events in 2017 and 2018, our report showed an increasing trend in emergent, critical and fatal injuries among older adult road users in rural, suburban, and urban areas. There is therefore a need for intensified and focused public health intervention in reducing older adult crash injury rates across the U.S. Preventing the interactions of several co-existing risk factors of crash occurrence is a strategy recommended by the Governors Highway Safety Administration [74]. For example, our study showed that night driving is associated with worse injury severity among older adult road users. However, intentionally increasing nighttime police presence and nighttime enforcement of speed limits on road sections and highways associated with high clusters of crash occurrence may reduce crash occurrence at night. Preventing the interaction effect of risk factors requires improved data collection and the creation of more sensitive spatial and non-spatial models. Additionally, there is a need to extend substance use educational intervention to non-conventional research settings where older adults commonly gather. Such locations include medical clinics, senior centers, retirement communities, places of worship, and parks and recreation centers. Achieving behavioral change in alcohol and illicit drug use among older adults requires identifying motivators for change, using positive messaging techniques, encouraging peer support, and exercising patience [83,84]. Furthermore, primary care providers should educate older adults on the harm of driving when given prescription drugs.

Our findings have a number of practical implications. First, there is a need for health education campaigns that target older adults and their communities. Increasing awareness about the potential risks associated with substance use, even in older age, can lead to more informed choices. Secondly, healthcare providers, especially those in primary care settings, can use this information to prioritize screening and early identification of older adults at risk of substance use-related crash injuries. Despite no significant difference in substance use among older adults living in rural, suburban, and urban areas, injury severity is worse among those living in rural and suburban areas. There is, therefore, a need for continued government funding for hospitals, healthcare facilities, and EMS systems in rural and suburban areas to allocate resources strategically to facilitate rapid response and care of crash injuries. Since older adults might have different social dynamics and support systems, community-based programs can play a significant role. These programs could include support groups, social activities, and initiatives that address both substance use and injury prevention. Furthermore, in rural and suburban areas, transportation and access to emergency medical services could be challenging. The federal, state, and local government as well as community leadership should regularly assess the community-level transportation needs and prioritize measures that will improve transportation options and safety for their older populations.

This study has its limitations. As a cross-sectional design, causal inferences cannot be made. We were unable to control for other risky driving behaviors such as the non-use of seatbelts, distracted driving, and speeding because these variables were not captured in the NEMSIS. We did not adjust for the level of certification of crash scene EMS staff since this information is not part of the publicly released data from the NEMSIS. Substance use, a heterogeneous term for alcohol, marijuana, narcotics, stimulants, depressants, benzodiazepines, and other illicit drugs is inconsistently screened across the U.S. Substance use screening differs across states with substantial under-reporting of drug use while driving [85], falsely lowering the effect size we report. The gold standard for a diagnosis of substance use remains a serological assessment [86]. However, with some cases of substance use identified by self-reported measures and the presence of paraphernalia of alcohol and drugs, the possibility of misclassification bias is likely. Misclassification of the outcome measure is less likely since injury severity classification was based on a complex matrix and applied by trained EMS staff. Despite these limitations, this study has several strengths, which include the generalizability of this study to older adults who sustain injuries across the U.S. Also, this is one of the few studies that assess the rural-urban association of substance use and crash injury severity among older adult road users. The awareness of the association between substance use and crash injury severity as well as the rural-urban differences will improve interventions aimed at reducing crash involvement of older adult road users.

## 5. Conclusion

Substance use is associated with worse crash injury severity among older adult road users. Despite no significant difference in rural-urban proportions of substance use among older adults, emergent injuries increase from urban to rural areas. With the increasing trends in critical and fatal crash injuries among older adults, there is an urgent need for increased community awareness of the risks associated with substance use among older adult road users. Also, reversing the uptrend pattern in substance use-related emergent, critical, and fatal crash injuries might require increased screening and identifying older adults who are at risk of substance use-related crash involvement, and increased healthcare resource allocation that will strengthen the emergency response systems, especially in rural and suburban areas.

## Supporting information

S1 Checklist STROBE statement—checklist of items that should be included in reports of observational studies. (DOCX)
