# Supplementary material for: Substance use and pre-hospital crash injury severity among U.S. older adults: A five-year national cross-sectional study
Source: PLoS One. 2023 Oct 25;18(10):e0293138. doi: 10.1371/journal.pone.0293138 (PMC10599556; doi:10.1371/journal.pone.0293138)
Supplement: S1 Checklist — (DOCX) [file pone.0293138.s001.docx]

STROBE Statement—checklist of items that should be included in reports of observational studies

|  | Item No. | Recommendation | Page  No. | Relevant text from manuscript |
| --- | --- | --- | --- | --- |
| **Title and abstract** | 1 | (*a*) Indicate the study’s design with a commonly used term in the title or the abstract | 1 | “Substance Use and Pre-Hospital Crash Injury Severity Among U.S. Older Adults: A Five-Year National Cross-sectional Study” |
|  |  | (*b*) Provide in the abstract an informative and balanced summary of what was done and what was found | 2 | “Methods: We pooled 2017 – 2021 cross-sectional data from the United States National Emergency Medical Service (EMS) Information System. We measured injury severity (low acuity, emergent, critical, and fatal) predicted by substance use, defined as self-reported or officer-reported alcohol and/or drug use. We controlled for age, sex, race/ethnicity, road user type, anatomical injured region, location (scene) of the injury, rurality/urbanicity, time of the day, and EMS response time. We performed a partial proportional ordinal logistic regression and reported the odds of worse injury outcomes (emergent, critical, and fatal injuries) compared to low acuity injuries, and the predicted probabilities by rurality/urbanicity.  Results: ….After controlling for patient, crash, and injury characteristics, substance use was associated with 35% increased odds of worse injury severity. Compared to urban areas, the predicted probabilities of emergent, critical, and fatal injuries were higher in rural and suburban areas.” |
| Introduction | | | |  |
| Background/rationale | 2 | Explain the scientific background and rationale for the investigation being reported | 4 | “It is unknown to what extent substance use is associated with injury severity among older adults. Additionally, it is not known how the relationship between substance use and crash injury severity among older adults differs across rural and urban areas. Identifying these regional differences may inform policies on safe driving, road infrastructural design, and targeted behavioral interventions for older adults. Assessing the risk of crash injury severity among older adults is important due to the increasing older US adult population,[30,31] and older licensed drivers [1].” |
| Objectives | 3 | State specific objectives, including any prespecified hypotheses | 4 | “This study, therefore, aims to assess the relationship between substance use and crash injury severity among older adults and the rural-urban differences that further define this problem.” |
| Methods | | | |  |
| Study design | 4 | Present key elements of study design early in the paper | 4 | “We conducted a cross-sectional analysis by pooling five years of data (2017 to 2021) from the National Emergency Medical Services (EMS) Information System (NEMSIS).” |
| Setting | 5 | Describe the setting, locations, and relevant dates, including periods of recruitment, exposure, follow-up, and data collection | 4 | “We conducted a cross-sectional analysis by pooling five years of data (2017 to 2021) from the National Emergency Medical Services (EMS) Information System (NEMSIS).” |
| Participants | 6 | (*a*) *Cohort study*—Give the eligibility criteria, and the sources and methods of selection of participants. Describe methods of follow-up  *Case-control study*—Give the eligibility criteria, and the sources and methods of case ascertainment and control selection. Give the rationale for the choice of cases and controls  *Cross-sectional study*—Give the eligibility criteria, and the sources and methods of selection of participants | 5 | “Between 2017 and 2021, 157,115,593 persons were managed following an EMS activation (Figure 1). We identified the older adult population (age 65 years and older) (n=58,272,048). We further restricted the population to age 65 years and older road users that sustained motor vehicle crash injuries using the International Classification of Disease version 10 (ICD-10) codes V00 to V79 (n= 489,565).” |
|  |  | (*b*) *Cohort study*—For matched studies, give matching criteria and number of exposed and unexposed  *Case-control study*—For matched studies, give matching criteria and the number of controls per case |  | NA |
| Variables | 7 | Clearly define all outcomes, exposures, predictors, potential confounders, and effect modifiers. Give diagnostic criteria, if applicable | 5-8 | Please see sections 2.3., 2.4., and 2.5. |
| Data sources/ measurement | 8* | For each variable of interest, give sources of data and details of methods of assessment (measurement). Describe comparability of assessment methods if there is more than one group | 5-8 | Please see sections 2.3., 2.4., and 2.5. |
| Bias | 9 | Describe any efforts to address potential sources of bias | 8 | “We performed multiple imputations for missing data, using the multiple imputations with chained equation (MICE) after justifying that missingness was at random [43]. Additionally, NEMSIS had advised researchers not to assume that missingness in the NEMSIS data is “Not Missing at Random”,[44] further stressing the need to perform some measures of missing data analysis whenever such missingness is encountered.” |
| Study size | 10 | Explain how the study size was arrived at | 5 | Please see section 2.2 |

Continued on next page

| Quantitative variables | 11 | Explain how quantitative variables were handled in the analyses. If applicable, describe which groupings were chosen and why | 5-8 | Please see sections 2.3., 2.4., and 2.5. |
| --- | --- | --- | --- | --- |
| Statistical methods | 12 | (*a*) Describe all statistical methods, including those used to control for confounding | 8 | “We performed unadjusted and adjusted partially proportional ordinal logistic regression [47] to assess the odds of worse injury outcomes – (critical, emergent, and fatal injuries) and computed the predicted probabilities of substance use-associated injury severity.” |
|  |  | (*b*) Describe any methods used to examine subgroups and interactions | 8 | “Also, we performed the interaction analysis between substance use and rurality/urbanicity and we reported the predicted probabilities of each substance use-related injury severity category in rural, suburban, and urban areas.” |
|  |  | (*c*) Explain how missing data were addressed | 8 | Please see sections 2.6. |
|  |  | (*d*) *Cohort study*—If applicable, explain how loss to follow-up was addressed  *Case-control study*—If applicable, explain how matching of cases and controls was addressed  *Cross-sectional study*—If applicable, describe analytical methods taking account of sampling strategy | NA |  |
|  |  | (*e*) Describe any sensitivity analyses | NA |  |
| Results | | | | |
| Participants | 13* | (a) Report numbers of individuals at each stage of study—eg numbers potentially eligible, examined for eligibility, confirmed eligible, included in the study, completing follow-up, and analysed | 5 | “Between 2017 and 2021, 157,115,593 persons were managed following an EMS activation (Figure 1). We identified the older adult population (age 65 years and older) (n=58,272,048). We further restricted the population to age 65 years and older road users that sustained motor vehicle crash injuries using the International Classification of Disease version 10 (ICD-10) codes V00 to V79 (n= 489,565). We excluded cases whose substance use status was coded as "not applicable" (n=19,519; 4% of 489,565). Thereafter, we excluded cases whose injury status was not reported (n=213,897; 45.5% of 470,046). These unreported cases represent patients who either canceled the 9-1-1 call, refused care, or were evaluated but no treatment or transport was required. Also, we performed a listwise deletion for cases whose missingness was less than one percent (n=1,203; 0.5% of 256,149) and when the crash response time was greater than 60 minutes (n=1,103; 0.4% of 256,149). We excluded cases whose EMS response time exceeded 60 minutes, consistent with an earlier study [26]. These outlier cases are typically associated with unique environmental conditions such as tornadoes [34-36]. The final analytic data, therefore, was a total of 253,933 older adult road users who sustained motor vehicle injuries.” |
|  |  | (b) Give reasons for non-participation at each stage | NA |  |
|  |  | (c) Consider use of a flow diagram | 5 | Please see Figure 1: Data selection steps using the 2017 to 2021 National Emergency Medical Service (EMS) Information System database. |
| Descriptive data | 14* | (a) Give characteristics of study participants (eg demographic, clinical, social) and information on exposures and potential confounders | 9 | “The majority of the population was between 65 and 74 years (62%), female (51%), non-Hispanic Whites (72%), and car occupants (76%). Thirty-six percent of the sample population sustained injuries to the chest and back. |
|  |  | (b) Indicate number of participants with missing data for each variable of interest | 7-8 | Please see “2.6. Handling of Missing Data” |
|  |  | (c) *Cohort study*—Summarise follow-up time (eg, average and total amount) | NA |  |
| Outcome data | 15* | *Cohort study*—Report numbers of outcome events or summary measures over time | NA |  |
|  |  | *Case-control study—*Report numbers in each exposure category, or summary measures of exposure | NA |  |
|  |  | *Cross-sectional study—*Report numbers of outcome events or summary measures | 9-10 | “Substance use was identified in approximately 3% of the sample population with cases of only alcohol or only drug impairments being 2.9% and 0.4%, respectively” |
| Main results | 16 | (*a*) Give unadjusted estimates and, if applicable, confounder-adjusted estimates and their precision (eg, 95% confidence interval). Make clear which confounders were adjusted for and why they were included | 16-17 | “Table 3: Unadjusted and adjusted odds ratio of worse injury severity (critical, emergent, death vs. low acuity) associated with the demographic, crash, injury, and substance use characteristics among older adults” |
|  |  | (*b*) Report category boundaries when continuous variables were categorized | 7 | “Age was measured as a three-level categorical variable (65 – 74 years, 75 – 84 years, and 85 years and older) while sex was measured as a binary variable.” |
|  |  | (*c*) If relevant, consider translating estimates of relative risk into absolute risk for a meaningful time period | NA |  |

Continued on next page

| Other analyses | 17 | Report other analyses done—eg analyses of subgroups and interactions, and sensitivity analyses | 17 | “The predicted probability of substance use-associated emergent injury was 32.0% (95% CI: 30.9 - 33.0) and the predicted probability increased step-wisely from urban (31.2%; 95% CI: 30.2 - 32.3) to suburban (33.5; 95% CI: 29.8 - 37.1) and rural areas (36.5%; 95% CI: 33.0 - 39.9) (p<0.001) (Figure 3).” |
| --- | --- | --- | --- | --- |
| Discussion | | | | |
| Key results | 18 | Summarise key results with reference to study objectives | 18 | “While substance use did not differ significantly between rural and urban settings, the likelihood of emergent, critical, and fatal injuries was significantly higher in rural/suburban areas compared to urban settings. Additionally, we report that four out of ten older road users involved in crash events who use alcohol or drugs are more likely to have emergent, critical, or fatal injuries and this likelihood is disproportionately higher in suburban and rural areas.” |
| Limitations | 19 | Discuss limitations of the study, taking into account sources of potential bias or imprecision. Discuss both direction and magnitude of any potential bias | 20 | “This study has its limitations. As a cross-sectional design, causal inferences cannot be made. We were unable to control for other risky driving behaviors such as the non-use of seatbelts, distracted driving, and speeding because these variables were not captured in the NEMSIS. We did not adjust for the level of certification of crash scene EMS staff since this information is not part of the publicly released data from the NEMSIS.” |
| Interpretation | 20 | Give a cautious overall interpretation of results considering objectives, limitations, multiplicity of analyses, results from similar studies, and other relevant evidence | 21 | “Substance use is associated with worse crash injury severity among older adult road users. Despite no significant difference in rural-urban proportions of substance use among older adults, emergent injuries increase from urban to rural areas. Increasing trends in critical and fatal older adult injuries should motivate urgent public health interventions.” |
| Generalisability | 21 | Discuss the generalisability (external validity) of the study results | 15 | “Despite these limitations, this study has several strengths, which include the generalizability of this study to older adults who sustain injuries across the U.S” |
| Other information | |  | | |
| Funding | 22 | Give the source of funding and the role of the funders for the present study and, if applicable, for the original study on which the present article is based | 1 | “The authors report no relevant disclosures. The author received no specific funding for this work.” |

*Give information separately for cases and controls in case-control studies and, if applicable, for exposed and unexposed groups in cohort and cross-sectional studies.

**Note:** An Explanation and Elaboration article discusses each checklist item and gives methodological background and published examples of transparent reporting. The STROBE checklist is best used in conjunction with this article (freely available on the Web sites of PLoS Medicine at http://www.plosmedicine.org/, Annals of Internal Medicine at http://www.annals.org/, and Epidemiology at http://www.epidem.com/). Information on the STROBE Initiative is available at www.strobe-statement.org.
